# Co-delivery of miR-29b and germacrone based on cyclic RGD-modified nanoparticles for liver fibrosis therapy

**DOI:** 10.1186/s12951-020-00645-y

**Published:** 2020-06-08

**Authors:** De Ji, Qiaohan Wang, Qi Zhao, Huangjin Tong, Mengting Yu, Meng Wang, Tulin Lu, Chengxi Jiang

**Affiliations:** 1 School of Pharmacy, Nanjing University of Chinese Medicine, Nanjing, 210023 China; 2 Molecular Pharmacology Research Center, School of Pharmaceutical Science, Wenzhou Medical University, Wenzhou, 325035 China; 3 Biomedical Collaborative Innovation Center of Zhejiang, Wenzhou, 325035 China; 4 Affiliated Hospital of Integrated Traditional Chinese and Western Medicine, Nanjing University of Chinese Medicine, Nanjing, 210028 China

**Keywords:** Hepatic stellate cells, Germacrone, miR-29b, cRGDfK, Liver fibrosis therapy

## Abstract

Hepatic stellate cells (HSCs) were activated and secreted excessive amounts of extracellular matrix (ECM) proteins during pathogenetic progress of liver fibrosis. Germacrone (GMO) and miR-29b can play an important role in inhibiting growth of HSCs and production of type I collagen. GMO and miR-29b were co-encapsulated into nanoparticles (NPs) based on poly(ethylene glycol)-block-poly(lactide-co-glycolide) (PEG-PLGA). Then, NPs were modified with cyclic RGD peptides (cRGDfK). cRGDfK is an effective ligand to bind integrin α_v_β_3_ and increase the targeting ability for fibrotic liver. GMO- and miR-29b-loaded NPs exhibited great cytotoxicity to activated HSCs and significantly inhibited production of type I collagen. Liver fibrosis model of mice was induced by administration of carbon tetrachloride. Great targeting ability was achieved in liver fibrotic mice treated with cRGD-modified NPs. Significant ant-fibrotic effects have been presented based on hematoxylin and eosin (H&E), Masson and Sirius Red staining results of liver tissues collected from mice treated with drug-loaded NPs. All these results indicate GMO- and miR-29b-loaded cRGD-modified NPs have the potential for clinical use to treat liver fibrosis.

## Introduction

Liver fibrosis has been serious health problem with high worldwide dominance and poor prognosis [1–4]. Liver fibrosis is a responsive process of virtually all forms of chronic liver injury, including hepatitis infection, excess alcohol consumption, and metabolic disorders [5–7]. With the deepening of liver fibrosis, it may result in cirrhosis, organ failure, and even hepatocellular carcinoma [8]. In this process, hepatic stellate cells (HSCs) were activated by factors released from damaged hepatocytes and secreted excessive extracellular matrix (ECM). ECM contains different components, including collagen, proteoglycans, fibronectin, and hyaluronic acid, dominated by collagen I [9, 10]. Thus, activated HSCs become important targets of anti-fibrotic therapy and the therapeutic effect can be reflected by the level of collagen I. After decades of efforts for basic and clinical trials, several anti-fibrotic agents are currently in clinical trials [11]. However, no drugs have yet emerged as effective anti-fibrotic agents, because medicinal candidates cannot specifically target HSCs and are frequently toxic to parenchymal cells [12]. Thus, there is still an urgent need to find new drugs and develop effective anti-fibrotic therapies [4].

Germacrone (GMO) is one of the main bioactive components in the traditional Chinese medicine Rhizoma curcuma [13]. Fufang Biejia Ruangan Pill (FFBJ) has been approved by the China State Food and Drug Administration to use as the first anti-fibrosis drug [14]. Noteworthily, GMO is an important component and has been demonstrated inducing G2/M cell cycle arrest and apoptosis through inhibition of JAK2/STAT3 signal pathway against human hepatoma cell lines [13]. However, the anti-fibrosis effect of GMO has not been reported yet. In this study, GMO was selected as the potential drug and encapsulated into NPs to evaluate its anti-fibrosis effect.

MicroRNAs (miRNAs) are receiving increasing attention for the management of different diseases. miRNAs are endogenous small noncoding RNAs and act as the regulator for RNA expression [11]. miRNAs exhibit great safety and potential for disease treatment due to their endogenous nature and biological functions. Besides, miRNAs have already been exploited for the treatment of liver fibrosis [15–17]. miRNAs play a crucial role in the initiation and progression of liver fibrosis. As reported, miR-29b family is significantly down-regulated during the activation of HSCs and progression of liver fibrosis [18]. miR-29b targets several pro-fibrotic genes like collagen type I & IV, c-MYC, platelets derived growth factor beta (PDGF-β), and PI3K/AKT through their 3′-UTR regions [19]. Notably, miR-29b down-regulates phosphorylation of protein kinase B (AKB) which is involved in growth and proliferation, adhesion, migration, and collagen production by HSCs. Transforming growth factor beta 1 (TGF-β1) up-regulated in liver fibrosis and known to down-regulate miR-29b through SMAD3 pathway. Therefore, liver fibrosis can possibly be attenuated by restoring intracellular levels of miR-29b to inhibit fibrogenic signaling at multiple levels and expression of ECM proteins.

In recent years, drug delivery systems have attracted increasing attention because of their unique advantages, like tissue or cell targeting [20–22], combined therapy [23, 24] and imaging [25–28]. Drug delivery system strategies have showed great potential for cancer treatment by improving traditional chemotherapy [29, 30] and incorporating novel therapy technology [31–34]. Although some drug delivery systems have been developed for the treatment of liver fibrosis (Table 1) [10, 12, 35–41], most of the work involved complex material synthesis or nanocarrier construction, which makes them difficult for massive generation and hence reduces their potential for clinical application. Besides, few new therapeutic agents were studied as the potential options of liver fibrosis therapy. Therefore, it is really still worth exploring new anti-fibrotic agents and paying more attention for clinical application. In this study, GMO was studied as a potential anti-fibrotic agent for the first time and co-loaded with miR-29b into NPs based on commercial block polymers poly(ethylene glycol)-block-poly(lactide-co-glycolide) (PEG-PLGA) to establish a drug delivery system for liver fibrosis therapy. To enhance the targeting ability, cRGDfK was connected with the carboxyl groups of NPs by amido bonds. Drug-loaded NPs were characterized by particle size, Zeta potential, drug loading, miRNA complexation as well as stability and transfection efficiency, and evaluated for treating liver fibrosis. Our results suggest a new approach for co-delivery of miRNAs and small hydrophobic molecules by biodegradable polymeric NPs.

**Table 1:** Recent development of drug delivery systems for liver fibrosis therapy

Nanocarriers	Targeted molecules	Drugs	Reference
liposomes	Vitamin A	Imatinib	[10] (2017)
PLGA-PSPE-PEG NPs	Vitamin A	Silibinin & siCol1α1	[12] (2018)
CaP@BSA NPs	/	TSG-6	[35] (2020)
HA nanomicelles	Hyaluronic acid	Silibinin	[36] (2019)
PEG-PLGA NPs	Vitamin A	Nilotinib	[37] (2019)
CS nanomicelles	Chondroitin sulfate	Doxorubicin & retinoic acid	[38] (2019)
Lipid nanoparticles	Vitamin A	siCol1α1 & siTIMP-1	[39] (2020)
Liposomes	cRGDyK	Vismodegib	[40] (2019)
Polymeric micelles	/	Polydatin	[41] (2020)

## Materials and methods

### Materials

PLGA_10k_-PEG_5k_-COOH was purchased from Huateng Pharma. Germacrone (GMO) was brought from J&K Scientific (China). miR-29b was supplied by Guangzhou RiboBio Co.,LTD. (China, 98%). Indocyanine green (ICG) was purchased from Tianjing HEOWNS Biochemical Technology Co., Ltd. (China, 98%). cRGDfK was brought from Hangzhou Peptide Biochem Co.,Ltd. (China, 97%). Poly(vinyl alcohol) (PVA, MW: 30000) was brought from Aladdin Biochemical Technology Co., Ltd. (China). *N*-Hydroxysuccinimide (NHS, China, 99%) and 1-(3-dimethylaminopropyl)-3-ethylcarbodiimide hydrochloride (EDC; China, 99%) were obtained from Sigma-Aldrich. 3-(4, 5-Dimethylthiazol-2-yl)-2, 5-diphenyltetrazolium bromide (MTT, China, 99%) and dihydrochloride (DAPI, China, 99%) was purchased from Sigma-Aldrich (Shanghai) Trading Co., Ltd. BCA protein assay kit was brought from Beyotime Biotechnology Co., Ltd. Carbon tetrachloride (CCl_4_, China, AR) was purchased from Energy Chemical. Organic solvents including chloroform and dimethyl sulfoxide (DMSO) were brought from Greagent (China, AR).

### Preparation and modification of NPs

The GMO- and miR-29b-loaded PEG-PLGA-COOH NPs (G/R-NPs) were prepared using the oil-in-water (O/W) emulsion method [42]. Briefly, PLGA-PEG-COOH (12.5 mg) was dissolved in 0.25 mL chloroform and mixed with 2.3 mg GMO in 15 μL chloroform under ultrasound. Then 15 μL miR-29b was added dropwise into the organic phase and mixed by sonication at 0 °C for 2 min. The solution was further dropwise added into 1.5 mL of 1% (wt/v) PVA and mixed by sonication for another 2 min. At last, 0.3% PVA (25 mL) was added and the mixture was stirred at room temperature overnight to evaporate the organic solvent. The obtained emulsion was washed by ultrafiltration for three times (MW 100 kD) at 4 °C and 1.25 mL PBS (pH 7.4) was added to absorb the NPs.

To modify cRGD onto the NPs, EDC (0.25 mg) and NHS (1.25 mg) were mixed with NPs suspension and stirred for 30 min. cRGD (1.25 mg) was added and the reaction were stirred at 4 °C overnight. The cRGD-modified G/R-NPs (G/R-RGD-NPs) were purified by ultrafiltration (MWCO 5000) for three times at 10000 rpm to remove EDC, NHS and unconjugated cRGD. Single drug loaded NPs were prepared and modified by the same method, abbreviated as G-RGD-NPs and R-RGD-NPs, and used in the following experiments.

To visually demonstrate the enhanced cellular uptake and targeting ability of cRGD-modified NPs, ICG-loaded NPs based on PEG-PLGA-COOH were prepared and modified with cRGD by the same procedure, abbreviated as ICG-NPs and ICG-RGD-NPs, respectively.

The colloidal stability of cRGD-modified PEG-PLGA NPs was evaluated by DLS in different conditions: water, saline and culture medium containing 10% fetal bovine serum (FBS). Briefly, NPs were incubated in different conditions and shaken slightly at 37 °C for a week. The particle size of NPs was measured by DLS every day. The experiment was performed for three times.

### Characterization of NPs

The size distribution and Zeta potential of blank NPs, G/R-NPs and G/R-RGD-NPs were measured by dynamic light scattering (DLS). The morphology of NPs was observed by transmission electron Microscopy (TEM, H7100). The TEM sample was prepared by the typical procedure as reported [43]. In brief, 10 μL NPs suspension was dropped onto carbon-coated copper grids. After stained for 5 min, negative staining with 2% phosphotungstic acid was used to enhance the contrast. After drying for 1 h at room temperature, NPs images were observed under TEM.

### Determination of drug loading content (DLC) and efficiency (DLE)

DLC of GMO in G/R-RGD-NPs was determined by high performance liquid chromatography (HPLC, Shimadzu, Japan). In brief, G/R-RGD-NPs were dissolved in methanol. Sonication was performed to destroy NPs. After filtration with 0.22 μm filter, the filtrate was analyzed by HPLC at excitation wavelength 210 nm. Mobile phase comprised of acetonitrile/phosphoric acid solution (50/50, v/v, pH 3.24) with the flow rate of 1.0 mL/min.

The DLC of miR-29b was measured spectrophotometrically. Briefly, after miR-29b-loaded NPs were prepared, the suspension was centrifuged and the liquid supernatant was collected to measure the amount of free miR-29b. The amount of encapsulated miR-29b is equal to the total amount of input minus the amount of free miR-29b. DLC and DLE were calculated as follows:

1 $$DLC \% = \frac{Amount\;of\;drugs\;entrapped\;in\;NPs}{Initial\;amount\;of\;drug\;added} \times 100 \%$$

2 $$DLE \% = \frac{Amount\;of\;drugs\;entrapped\;in\;NPs}{Total\;amount\;of\;NPs} \times 100 \%$$

### Drug release

The release profiles of G/R-RGD-NPs were investigated in PBS at 37 °C. Briefly, G/R-RGD-NPs (1 mg/mL) were added into a dialysis tube (MW 3500) and immersed in 10 mL mixture of PBS with 1% SDS (pH 7.4): methanol (60/40, v/v) in a brown bottle [44]. The bottle was then placed at 37 °C with mild shaking. At the predetermined time points during incubation, 1 mL release medium was taken out to measure drug content and the same volume of fresh release medium was added. The release medium was lyophilized and re-dissolved in methanol and filtrated through 0.22 μm filter before measurement.

### Cell culture

HSCs cells were cultured in DMEM supplemented with 10% fetal bovine serum (FBS), 100 units/mL penicillin and 100 μg/mL streptomycin. LO2 cells were maintained in RPMI-1640 media containing 10% FBS, 100 units/mL penicillin and 100 μg/mL streptomycin. All cells were incubated at 37 °C in a humidified 5% CO_2_ atmosphere.

### Cellular uptake

Intracellular uptake of NPs was evaluated by fluorescence imaging. Activated HSCs were seeded into 6-well plates with the density of 3.0 × 10^5^ cells/well. After 12 h incubation, cells were treated with ICG-NPs and ICG-RGD-NPs for 4 h. In addition, to further confirm cRGD enhanced cellular uptake, cells were pretreated with free cRGD for 20 min to block α_v_β_3_ before ICG-RGD-NPs was added. Subsequently, cells were washed with PBS for three times and fixed with 4% paraformaldehyde for 20 min. After the nuclei were stained with DAPI solution for 15 min, the cells were exposed to a fluorescence microscopy. To survey the time-dependent intracellular accumulation, cells were seeded and incubated as the procedure mentioned above, except cells were treated with ICG-RGD-NPs and incubated for another 2 h, 4 h, 8 h and 12 h. Cells were fixed and stained as the above procedure and exposed to a fluorescence microscopy.

### Cytotoxicity

MTT assay was performed to test the cytotoxicity of blank NPs, free GMO, G-RGD-NPs, R-RGD-NPs and G/R-RGD-NPs. First, cells were seeded in 96-well plates at the density of 5000 cells/well. After incubation for 12 h, cells were exposed to 200 μL of fresh culture medium containing various formulations of drugs and blank NPs for another 72 h. After that, the culture medium was replaced with 100 μL of fresh medium and 20 μL of MTT (5 mg/mL) solution was added afterwards. After incubation for another 4 h, the medium was removed and 200 μL DMSO was added. After the crystals were completely dissolved, a microplate reader was used to measure the optical density (OD) at 570 nm. The cell viability was obtained using the following equation:

3 $$Cell\; viability \% = \frac{{OD_{sample} - OD_{blank} }}{{OD_{control} - OD_{blank} }} \times 100 \%$$

where OD_sample_ represents the OD value of cells incubated with various drug formulations, OD_control_ represents the OD value of cells incubated with complete culture alone, and OD_blank_ represents the OD value of complete culture without cells.

### Immunofluorescence and immunohistochemistry

HSCs were seeded on glass coverslips and incubated for 24 h. Cells were treated with free GMO, G-RGD-NPs, R-RGD-NPs and G/R-RGD-NPs for 6 h. After that, the cells were washed with PBS for 15 min and fixed in 4% paraformaldehyde for 30 min. Cells were then washed and permeabilized with 0.1% (v/v) Triton X-100 for 20 min and washed with PBS for 15 min. Then, cells were immersed into 3% H_2_O_2_ solution and incubated for 10 min at room temperature to block endogenous peroxidase. After washing with PBS for 15 min, 5% (v/v) bovine serum albumin (BSA) was used as blocking solution for 20 min, followed incubating cells with primary antibodies in blocking solution overnight at 4 °C. Cells were then washed with blocking solution and incubated with fluorophore-labeled secondary antibodies for 50 min at room temperature. After another washing step, the cells were stained with DAPI for 10 min. After a final washing step, fluorescence images were observed by a fluorescence microscopy. Immunohistochemical analysis of cells was performed by the method mentioned above, except secondary antibodies used instead of fluorophore-labeled secondary antibodies.

### Western blot

Activated HSCs were seeded in 6-well plates with a density of 3.0 × 10^5^ cells/well and incubated for 12 h. Cells were treated with different formulations for 24 h before western blot analysis. Cells or liver tissues were washed with PBS for three times, followed by protein isolation with radioimmunoprecipitation assay (RIPA) buffer and determination with a BCA protein assay kit. The cell lysates were then mixed with loading buffer, boiled buffer at 100 °C for 5 min, loaded on the wells of SDS-PAGE gel, transferred by electroporation to poly-(vinylidene difluoride) (PVDF) membrane, incubated with blocking buffer for 30 min at 37 °C, and cultured with primary antibodies recognizing type I Collagen as well as β-actin primary antibody overnight at 4 °C with shaking; secondary antibodies were added and incubated for 4 h at room temperature followed by analysis using Chemiluminescent substrate. Blots performed in quintuplicate were imaged and quantified using ImageJ software with densitometry analysis.

### Liver fibrosis mice model

Eight-week-old male C57BL/6 mice were purchased from Model Animal Research Center of Nanjing University and reared in specific pathogen-free conditions. The protocol was proved by the Committee on the Ethics of Animal Experiments of Nanjing Medical University. Mice were treated weekly with CCl_4_ at a dose of 100 mg/kg (prepared with olive oil 1:1 v/v). After 4 weeks, animals developed liver fibrosis as validated by pathological analysis.

### Biodistribution

Liver fibrosis models were established as described above. Healthy or fibrotic mice were randomly divided into three groups: ICG-NPs and ICG-RGD-NPs at an equivalent dose of 2 mg/kg ICG with saline as control. In 6 h, 12 h and 24 h later, mice were sacrificed and major organs including heart, liver, lung, spleen and kidney were collected. The tissue fluorescent images were obtained using a Maestro imaging system.

### Therapeutic efficiency

To determine the therapeutic effect, fibrotic mice were divided into five groups and administered intravenously with saline, free GMO, G-RGD-NPs, R-RGD-NPs and G/R-RGD-NPs twice a week for 2 weeks. The dosages of GMO and RNA were 8 mg/kg and 0.5 mg/kg, 16 mg/kg and 1 mg/kg and 32 mg/kg and 2 mg/kg, respectively. During this period, survival rate was 100% in all groups of mice. After treatment, mice were sacrificed and livers were harvested and stored for further evaluation.

### Hematoxylin–Eosin (H&E), masson and sirius red staining

To assess the therapeutic effects of various therapy formulations, liver tissues were collected from treated fibrotic mice or healthy mice and fixed with formalin solution (4%, w/v). H&E, Masson and Sirius Red staining were performed after dehydration, wax dipping, transparency, embedding and 4 μm section of liver tissues. Histological analysis was conducted using an optical microscope.

### Results and discussion

Given the overexpression of α_v_β_3_ on activated HSCs during liver injury, cRGD-modified NPs were prepared based on FDA approved poly(lactic-co-glycolic acid)-poly(ethylene glycol) (PLGA-PEG) and encapsulate miR-29b and GMO for targeted liver fibrosis therapy. This study aims to explore the target ability and therapeutic efficacy of G/R-RGD-NPs in animal models.

### Preparation and characterization of NPs

**Fig. 1:**
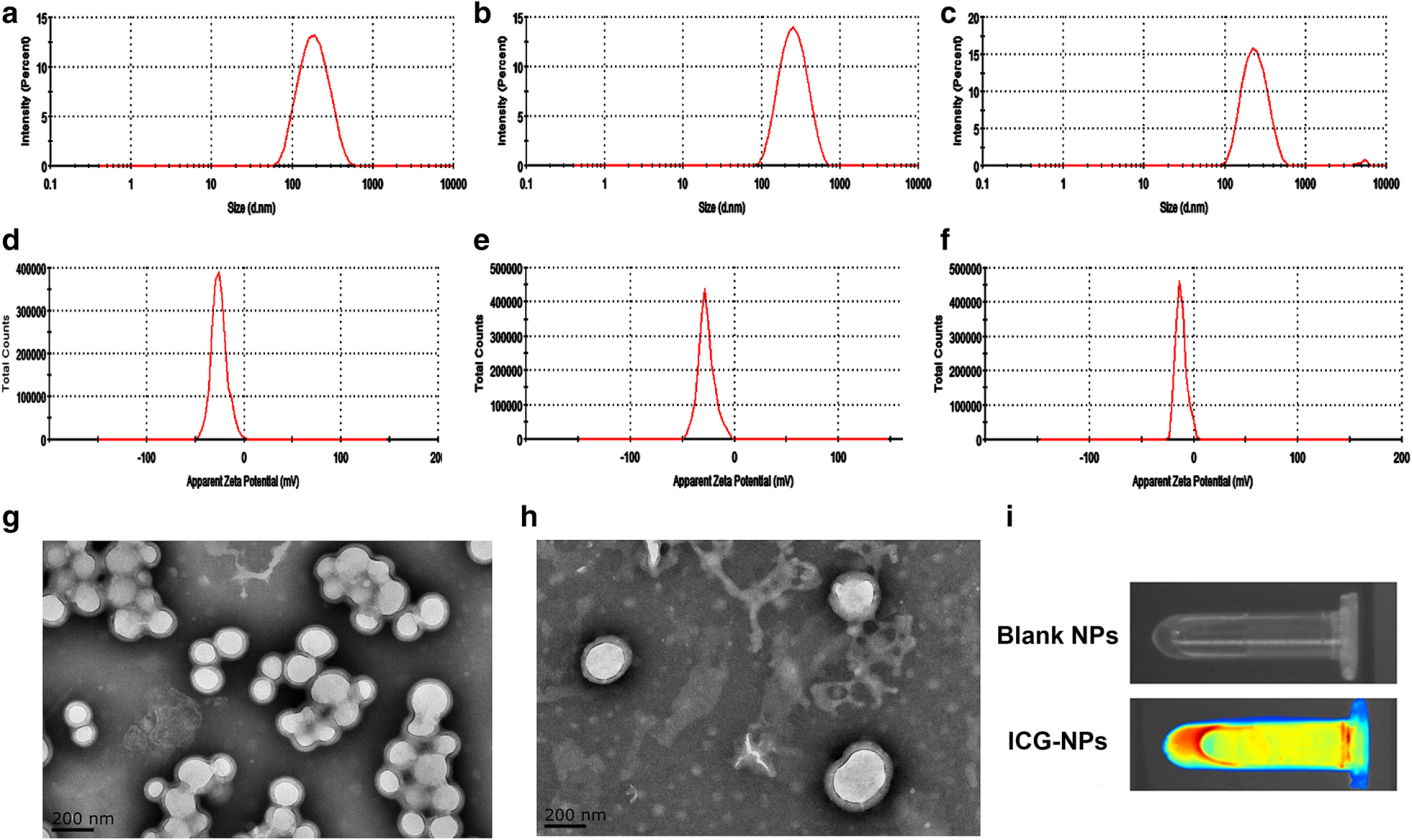
Size distribution of blank NPs **a**, G/R-NPs **b** and G/R-RGD-NPs **c** measured by DLS; Zeta potential of blank NPs **d**, G/R-NPs **e** and G/R-RGD-NPs **f** measured by DLS; TEM photos of G/R-NPs **g** and G/R-RGD-NPs **h**; photo of fluorescent images of blank NPs and ICG-RGD-NPs **i** using a Maestro imaging system

NPs were prepared by the oil-in-water (O/W) emulsion method. DLS results were shown in Fig. 1a–f including average size, polydispersity (PDI) and Zeta potential of blank NPs, G/R-NPs and G/R-RGD-NPs. All NPs exhibited uniform size distribution. Blank NPs displaced average size around 164.7 nm with PDI 0.168. After miR-29b and GMO were loaded, average size of NPs increased to be 231.2 nm with PDI 0.140 compared with blank NPs, while Zeta potential of G/R-NPs exhibited almost no change compared with blank NPs. The increased particles size was also observed in TEM results in Fig. 1g, h. All NPs showed negative surface charges, which was caused by carboxyl groups of PLGA-PEG-COOH. After cRGD was connected to the surface of NPs, G/R-RGD-NPs displaced average size of 227.2 nm with PDI 0.195. However, Zeta potential of G/R-RGD-NPs changed to be − 12.0 mV compared with G/R-NPs (− 27.0 mV), which proved that cRGD was successfully connected to the surface of NPs. The stability of cRGD-modified NPs was measured by DLS. As Additional file 1: Figure S3 depicted, cRGD-modified NPs can keep stable in water and saline, while the average size of cRGD-modified NPs in culture medium with 10% FBS showed a gradual increase.

DLC and DLE of GMO were measured as 2.5% and 13.3%, respectively. UV spectra of miR-29b in the supernatant and the initial amount of miR-29b were shown in Additional file 1: Figures S1A, B. After calculation, DLC and DLE of miR-29b were measured as 0.16% and 61.4%, respectively. It is worth noting that DLE of miR-29b was much larger than that of GMO, which is probably determined by the structure of hollow vesicle of NPs. Water-soluble miR-29b was encapsulated in the aqueous-favoring hollow, while lipophilic GMO was loaded in the hydrophobic layer.

The drug release of GMO from NPs was simulated in physiological environment (PBS with 1% SDS (pH 7.4): methanol (60/40, v/v)). As shown in Additional file 1: Figure S2, in the first 24 h, 77.6% of GMO was released, while only 6.1% of GMO was released in the next 24 h. However, almost no miR-29b was detected during 96 h (data not shown). In the simulated release condition, PEG-PLGA was not degraded and thus the NPs still protected encapsulated drugs in the aqueous phase.

### Cellular uptake and cytotoxicity

**Fig. 2:**
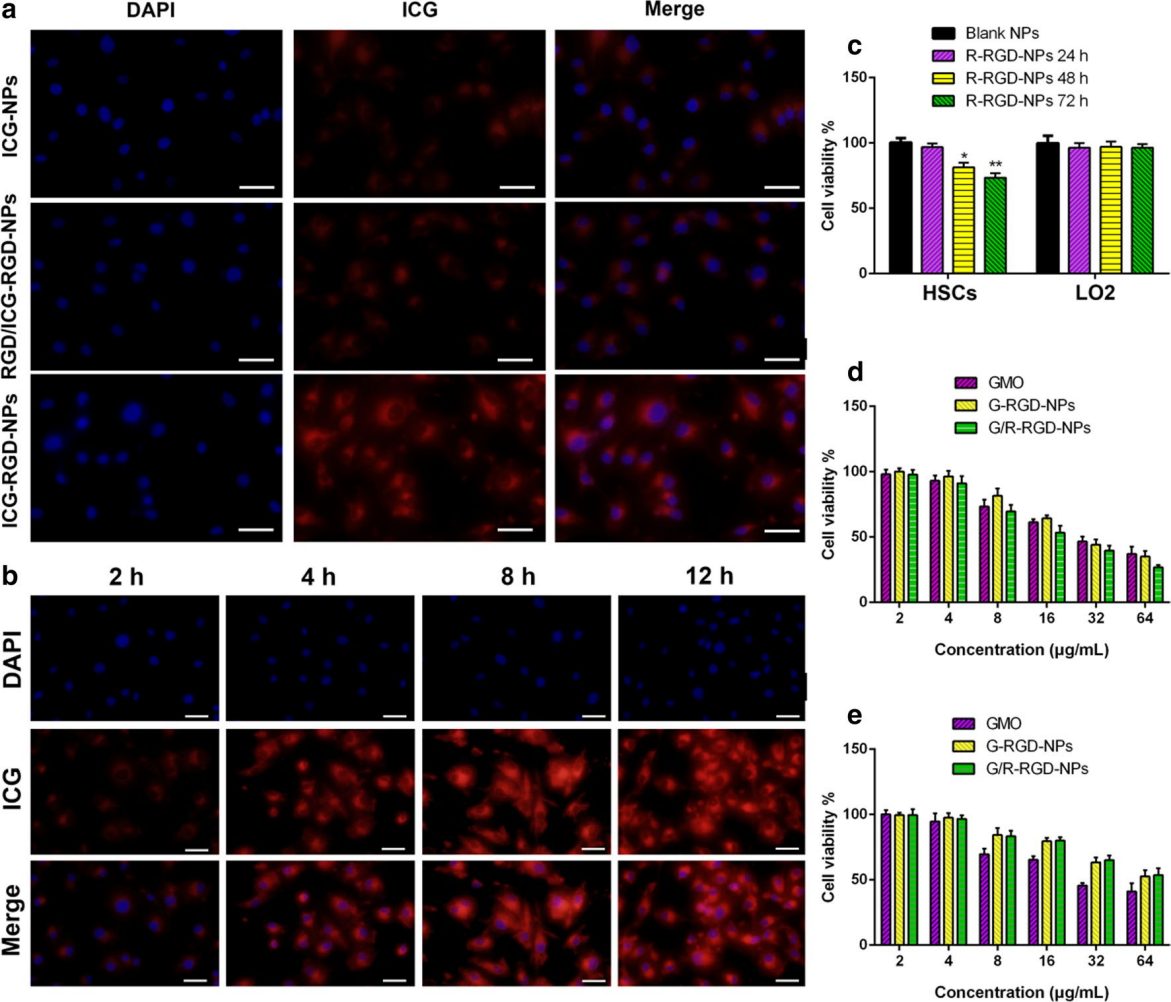
Fluorescent images of activated HSCs incubated with ICG-NPs and ICG-RGD-NPs with or without free cRGD pretreatment for 4 h **a**; fluorescent images of activated HSCs incubated with ICG-RGD-NPs for 2 h, 4 h, 8 h and 12 h **b**; cell viability of blank NPs (1 mg/mL) and R-RGD-NPs (1 mg/mL) against HSCs and LO2 for 24 h, 48 h and 72 h **c**; cell viability of free GMO, G-RGD-NPs and G/R-RGD-NPs against HSCs **d** and LO2 cells **e**. Scale bar: 20 μm

It has been reported that α_v_β_3_ was overexpressed in activated HSCs [45, 46]. Overexpression of α_v_β_3_ was observed on the activated HSCs compared with LO2 as shown in Additional file 1: Figure S4. Significantly increased green fluorescence was shown on activated HSCs. To demonstrate α_v_β_3_-mediated cellular uptake against activated HSCs, cells were treated with ICG-NPs and ICG-RGD-NPs with or without free cRGD pretreatment. As shown in Fig. 2a, cells treated with ICG-NPs displaced weak red fluorescence after cells incubated with different formulations for 4 h, while cells treated with ICG-RGD-NPs exhibited increased red fluorescence. These results were possibly explained by the fact that cRGD-modified NPs were able to be efficiently internalized into the cells via α_v_β_3_-mediated endocytosis. To further assess the specificity of binding of cRGD-modified NPs to activated HSCs, cells were pretreated with free cRGD to block α_v_β_3_ receptors. As expected, the fluorescence without cRGD pretreatment was much stronger than that with cRGD pretreatment, indicating that the uptake of cRGD-modified NPs can be competed out by excess free cRGD, which was consistent with a previous report [40].

To further demonstrate time-dependent accumulation of cRGD-modified NPs in activated HSCs, cells were treated with ICG-RGD-NPs for 2 h, 4 h, 8 h and 12 h. As depicted in Fig. 2b, activated HSCs exhibited increased fluorescence intensity after treated with ICG-RGD-NPs for 12 h. All these results indicated time-dependent accumulation of cRGD-modified NPs.

As shown in Fig. 2c, blank NPs exhibited no cytotoxicity against activated HSCs and LO2 even at a high concentration of 1 mg/mL. Cell viability of R-RGD-NPs was 78.3% and 89.4% after incubation for 72 h and 48 h, respectively, while cell viability of R-RGD-NPs was over 93% after incubation for 24 h. Besides, R-RGD-NPs exhibited almost no cytotoxicity against LO2 cells.

The cytotoxicity was evaluated after activated HSCs and LO2 were treated with free GMO, G-RGD-NPs and G/R-RGD-NPs. As shown in Fig. 2d, e, all GMO formulations displayed dose-dependent cytotoxicity against activated HSCs and LO2. G-RGD-NPs exhibited considerable cytotoxicity compared with free GMO against HSCs, while lower cytotoxicity than free GMO especially at high concentration of GMO against LO2. One of the possible reasons to explain these results is that α_v_β_3_ was overexpressed in activated HSCs, facilitating cRGD-modified NPs to be internalized into cells. Besides, it was worth noting that G/R-RGD-NPs exhibited higher cytotoxicity than other formulas at all tested concentrations against HSCs, while considerable cytotoxicity against LO2, which was in accordance with the results in Fig. 2c.

### Collagen secretion in activated HSCs

**Fig. 3:**
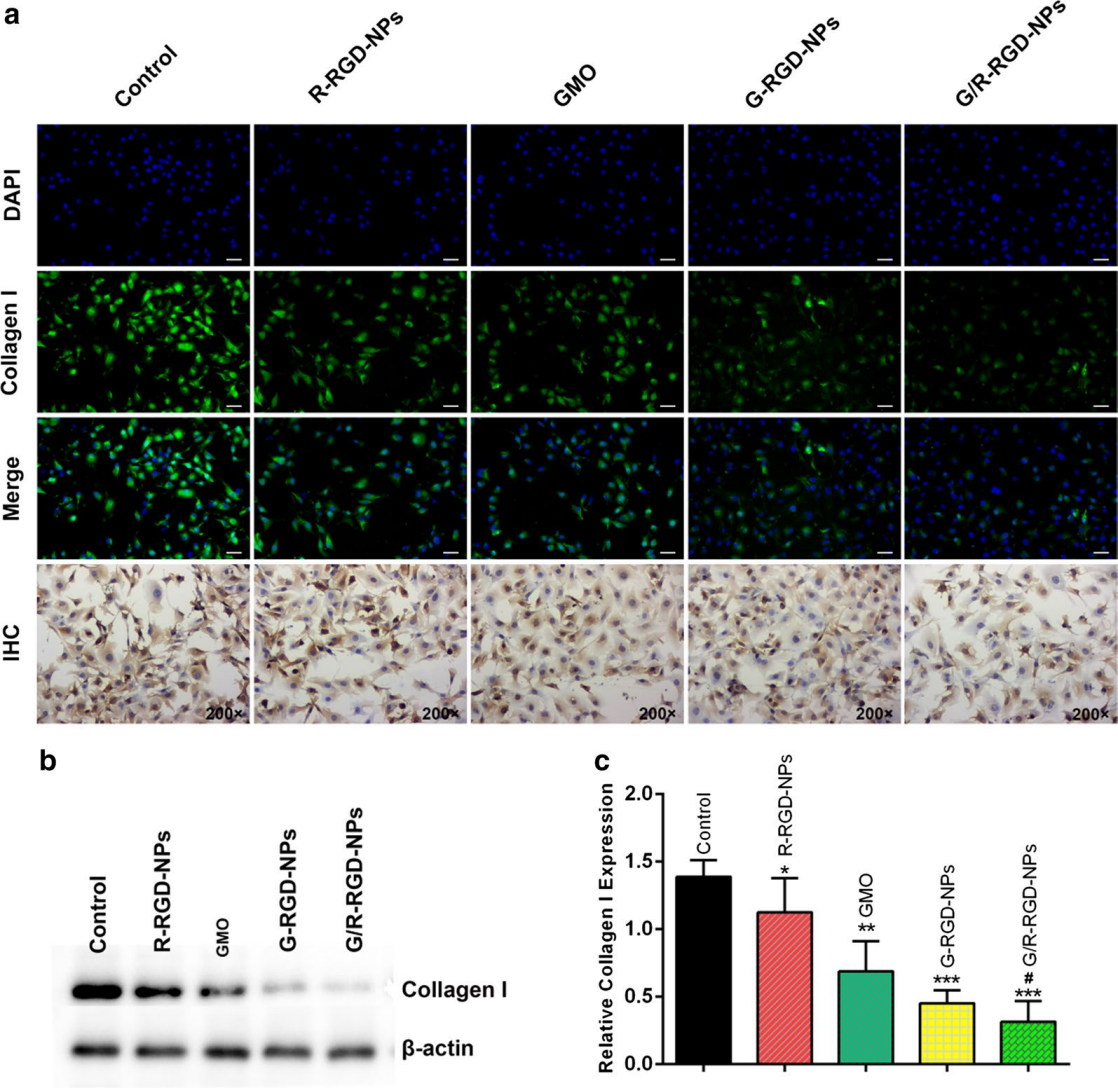
Immunofluorescence and immunohistochemistry of activated HSCs treated with different drug formulations **a**; collagen I expression of HSCs treated with different drug formulations **b**; quantitative analysis of type I collagen in activated HSCs **c**. (*p < 0.05, **p < 0.01 and ***p < 0.001 vs Control; ^#^*p *< 0.05 vs G-RGD-NPs)

Collagen plays a vital role in biological process. They served as a structural matrix for tissue maintenance, development and regeneration. Collagen is the most abundant protein in mammals and a main component of ECM. Normal tissue development involves dynamic collagen remodeling processes which was a balance between collagen production and degradation. If the balance was broken, it will cause structural and metabolic abnormalities of collagen and various pathological conditions, including liver fibrosis. Therefore, detecting collagen expression was significant to diagnose and attenuate liver fibrosis. After staining collagen I, the green fluorescence was lower than that of control group after cells were treated with different formulations in Fig. 3a. It can be easily observed that the green fluorescence was the least in activated HSCs treated with G/R-RGD-NPs, indicating enhanced inhibition of collagen production, which was also proved by the results of immunohistochemistry. The collagen abundance was further determined by Western blot as shown in Fig. 3b, c. It was found that collagen was significantly lower than other groups when cells were treated with G-RGD-NPs or G/R-RGD-NPs. Besides, G/R-RGD-NPs exhibited enhanced inhibition of expression of type I collagen compared with G-RGD-NPs, suggesting great potential of G/R-RGD-NPs for liver fibrosis therapy.

### Biodistribution and anti-fibrotic effect

**Fig. 4:**
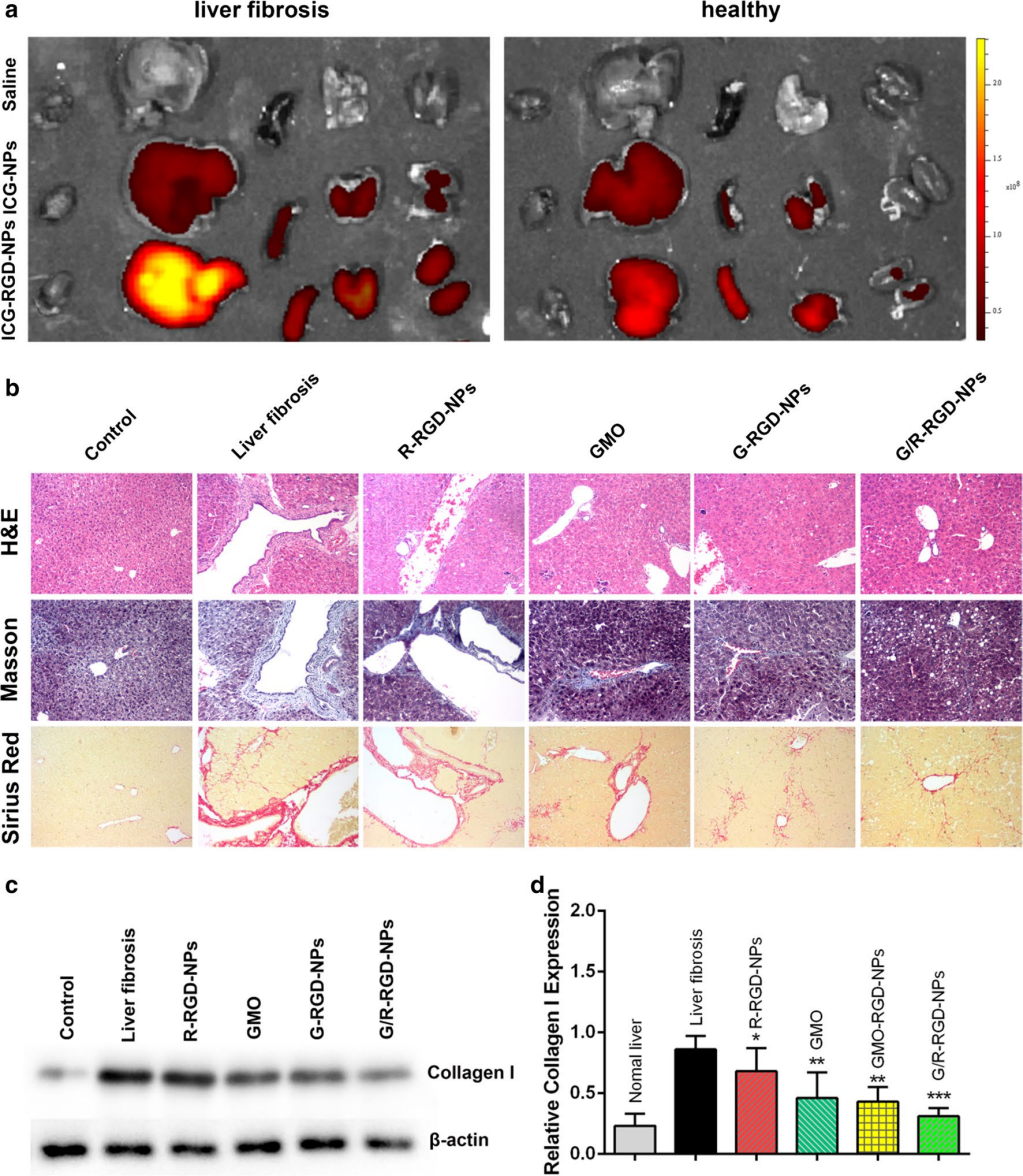
Biodistribution of ICG-NPs and ICG-RGD-NPs in mice of liver fibrosis and healthy mice after 24 h **a**; H&E, Masson and Sirius Red staining of livers collected from healthy and liver fibrotic mice **b**; collagen I expression of liver tissues from liver fibrotic mice treated with different drug formulations **c**; quantitative analysis of type I collagen in treated fibrotic livers **d**. (*p < 0.05, **p < 0.01 and ***p < 0.001 vs Control)

To evaluate biodistribution, fibrotic model mice were administrated with saline, ICG-NPs and ICG-RGD-NPs. Saline treatment was conducted as control groups. After 6 h, 12 h and 24 h, mice were sacrificed to collect isolated tissues, including heart, liver, spleen, lung and kidney. The ex vivo imaging of isolated tissues from liver fibrotic was carried out by an imaging system. ICG fluorescence was mainly concentrated in livers. ICG fluorescence was weak at 6 h, while it increased at 12 h (Additional file 1: Figure S5A). Notably, ICG-RGD-NPs exhibited significantly higher fluorescence than ICG-NPs at 12 h and 12 h (Fig. 4a).The fluorescence in spleen, lung and kidney increased at 24 h, indicating metabolism of the nanoparticles. Besides, stronger fluorescence was observed in livers from fibrotic or healthy mice treated with ICG-RGD-NPs than those from mice treated with ICG-NPs. Specifically, red fluorescence intensity was slightly higher in livers from healthy mice treated with ICG-RGD-NPs than that treated with ICG-NPs. All these results indicated that liver fibrotic mice model was established successfully and the targeting ability of cRGD-modified NPs was remarkably enhanced, which was mainly caused by two reasons: (1) HSCs activated and proliferated in the fibrotic liver thus increased binding sites for cRGD; (2) the fibrotic liver became dysfunctional and thus unable to clear NPs effectively.

To assess the therapeutic effects of various therapeutic formulations, the pathological examination was performed after mice were treated with different therapeutic agents by H&E, Masson and Sirius Red staining. To search the optimal dosages of GMO and miR-29b for the best therapeutic effect, low, medium and high dose of G/R-RGD-NPs were injected intravenously and the results were shown in Additional file 1: Figure S5B and C. All three groups of G/R-RGD-NPs exhibited dose-dependent anti-fibrosis effect and no obvious side effects can be detected. We chose medium dose for the following experiments. In Fig. 4b, H&E, Masson and Sirius Red staining revealed that treatment of CCl_4_ induced serious liver fibrosis compared with these of control group. And treatment of R-RGD-NPs displayed slightly anti-fibrotic effect in mice of liver fibrosis. GMO and G-RGD-NPs exhibited better therapeutic effect compared with R-RGD-NPs, and G/R-RGD-NPs exhibited the best anti-fibrotic effect in mice of liver fibrosis. Type I collagen from collected livers was measured as shown in Fig. 4c, d. Collagen I significantly decreased after mice with liver fibrosis were treated with all therapeutic formulations. GMO and G-RGD-NPs exhibited better inhibition of collagen I production than R-RGD-NPs. And G/R-RGD-NPs showed better inhibition of collagen I production than GMO and G-RGD-NPs.

## Conclusion

In summary, G/R-RGD-NPs have been established and applied in mice liver fibrosis model for the treatment of liver fibrosis. ICG-labeled RGD-NPs exhibited selective and time-dependent internalization in activated HSCs. Besides, the targeting ability of NPs was further enhanced by cRGD modification in liver fibrosis mice model. G/R-RGD-NPs exhibited higher cytotoxicity on HSCs and enhanced inhibition of protein production compared with free GMO, G-RGD-NPs and R-RGD-NPs. G/R-RGD-NPs also exhibited great anti-fibrosis effect on mice model. G/R-RGD-NPs may serve as a novel and effective clinical treatment option for liver fibrosis.

## Supplementary information

**Additional file 1: Figure S1.** UV spectra of miR-29b in the supernatant (A) and the initial amount of miR-29b (B). **Figure S2.** Standard curve of GMO (A); drug release of GMO from NPs simulated in physiological environment (PBS, pH 7.4) (B). **Figure S3.** Stability of cRGD-modified PEG-PLGA NPs in water, saline and culture medium with 10 % FBS. **Figure S4.** Overexpression of α_v_β_3_ against the activated HSCs compared with LO2. **Figure S5.** Biodistribution of ICG-NPs and ICG-RGD-NPs in mice of liver fibrosis after 6 h and 12 h (A). H&E, Masson and Sirius Red staining (B), collagen I expression (C) and quantitative analysis of collagen I (D) of livers collected from liver fibrotic mice treated with G/R-RGD-NPs of low, medium and high dose (*p < 0.05 *vs* Medium dose, #*p* < 0.05 *vs* Low dose, $*p* < 0.05 *vs* Liver fibrosis).

## Data Availability

The datasets used and/or analyzed during the current study are available from the corresponding author on reasonable request.

## Abbreviations

HSCs: Hepatic stellate cells
ECM: Extracellular matrix
GMO: Germacrone
NPs: Nanoparticles
PEG-PLGA: Poly(ethylene glycol)-block-poly(lactide-co-glycolide)
cRGDfK: Cyclic RGD peptides
H&E: Hematoxylin-eosin
miRNAs: MicroRNAs
G/R-NPs: GMO- and miR-29b-loaded PEG-PLGA-COOH NPs
G/R-RGD-NPs: cRGD-modified G/R-NPs
R-RGD-NPs: miR-29b-loaded cRGD-modified NPs
G-RGD-NPs: GMO-loaded cRGD-modified NPs
ICG-RGD-NPs: ICG-loaded cRGD-modified NPs
ICG-NPs: ICG-loaded NPs
DLC: Drug loading content
DLE: Efficiency
