# Supplementary material for: Co-delivery of miR-29b and germacrone based on cyclic RGD-modified nanoparticles for liver fibrosis therapy
Source: J Nanobiotechnology. 2020 Jun 8;18:86. doi: 10.1186/s12951-020-00645-y (PMC7281922; doi:10.1186/s12951-020-00645-y)
Supplement: Supplementary file 1 — Additional file 1: Figure S1. UV spectra of miR-29b in the supernatant (A) and the initial amount of miR-29b (B). Figure S2. Standard curve of GMO (A); drug release of GMO from NPs simulated in physiological environment (PBS, pH 7.4) (B). Figure S3. Stability of cRGD-modified PEG-PLGA NPs in water, saline and culture medium with 10 % FBS. Figure S4. Overexpression of αvβ3 against the activated HSCs compared with LO2. Figure S5. Biodistribution of ICG-NPs and ICG-RGD-NPs in mice of liver fibrosis after 6 h and 12 h (A). H&E, Masson and Sirius Red staining (B), collagen I expression (C) and quantitative analysis of collagen I (D) of livers collected from liver fibrotic mice treated with G/R-RGD-NPs of low, medium and high dose (*p < 0.05 vs Medium dose, #p < 0.05 vs Low dose, $p < 0.05 vs Liver fibrosis). [file 12951_2020_645_MOESM1_ESM.docx]

**Co-delivery of miR-29b and Germacrone Based on Cyclic RGD-modified Nanoparticles for Liver Fibrosis Therapy**

De Ji,*^,1,#^ Qiaohan Wang,^1,#^ Qi Zhao,^2,3^ Huangjin Tong,^4^ Mengting Yu,^1^ Meng Wang,^1^ Tulin Lu,*^,1^ and Chengxi Jiang*^,2,3^

^1^School of Pharmacy, Nanjing University of Chinese Medicine, Nanjing 210023, China

^2^ Molecular Pharmacology Research Center, School of Pharmaceutical Science

^3^Biomedical Collaborative Innovation Center of Zhejiang, Wenzhou, 325035 China

^4^Affiliated Hospital of Integrated Traditional Chinese and Western Medicine, Nanjing University of Chinese Medicine, Nanjing 210028, China

^#^These authors contributed equally to this work.

*Corresponding to: De Ji, School of Pharmacy, Nanjing University of Chinese Medicine, Nanjing 210023, China, E-mail: jidenucm@163.com. Tulin Lu, School of Pharmacy, Nanjing University of Chinese Medicine, Nanjing 210023, China, E-mail: [ltl209@163.com](mailto:ltl209@163.com). Chengxi Jiang, Life Sciences Institute, Wenzhou University, Wenzhou 325035, China, E-mail: jiangchengxi@126.com.


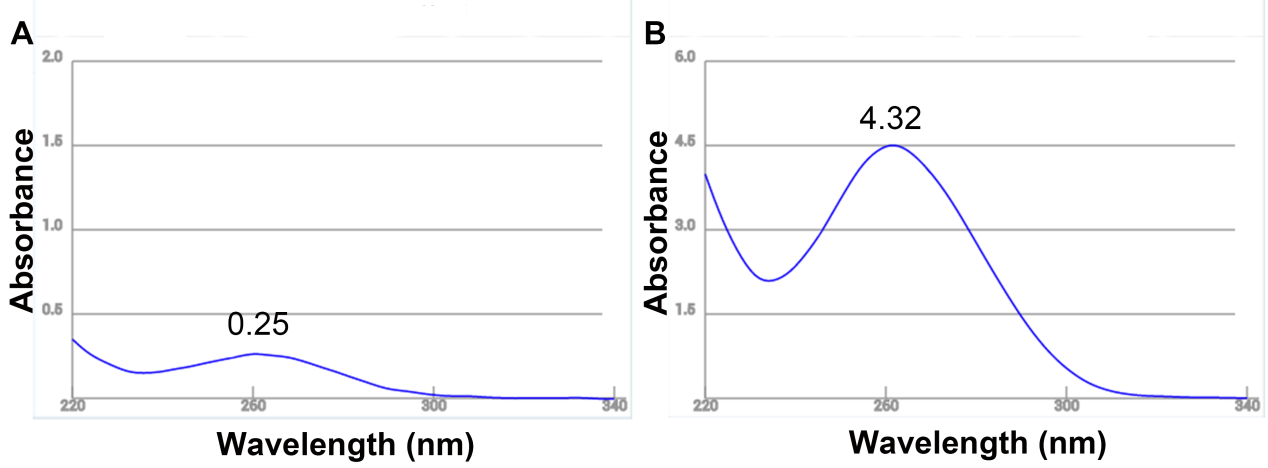


**Figure S1.** UV spectra of miR-29b in the supernatant (A) and the initial amount of miR-29b (B).


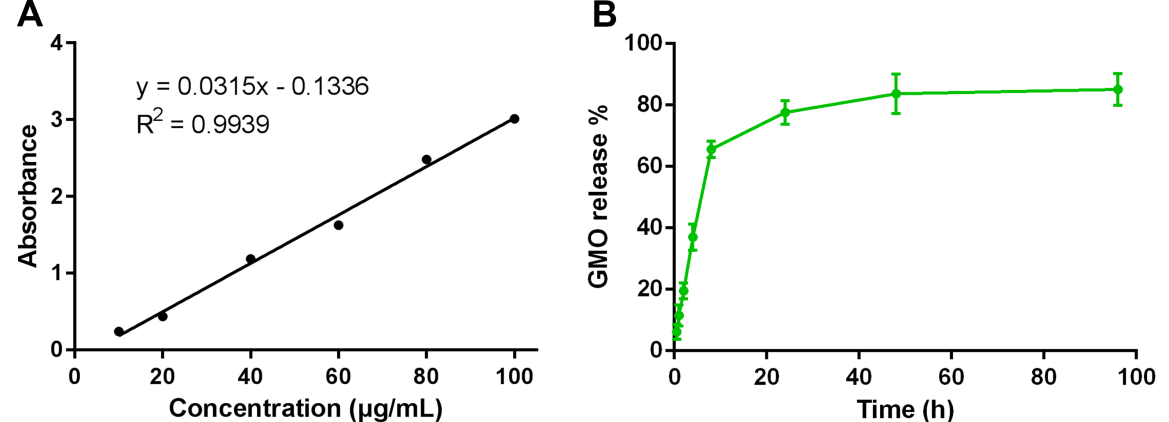


**Figure S2.** Standard curve of GMO (A); drug release of GMO from NPs simulated in physiological environment (PBS, pH 7.4) (B).


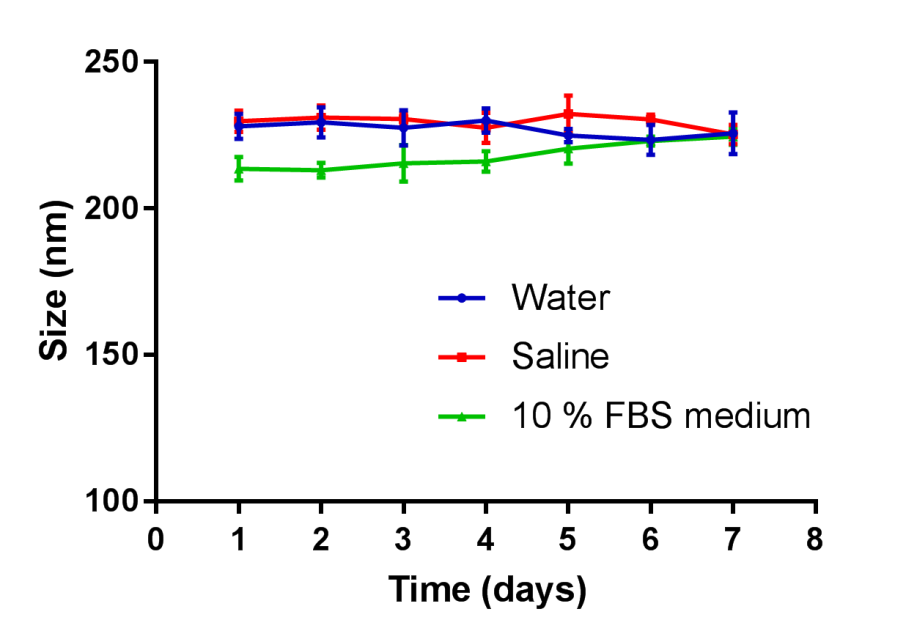


**Figure S3.** Stability of cRGD-modified PEG-PLGA NPs in water, saline and culture medium with 10 % FBS.


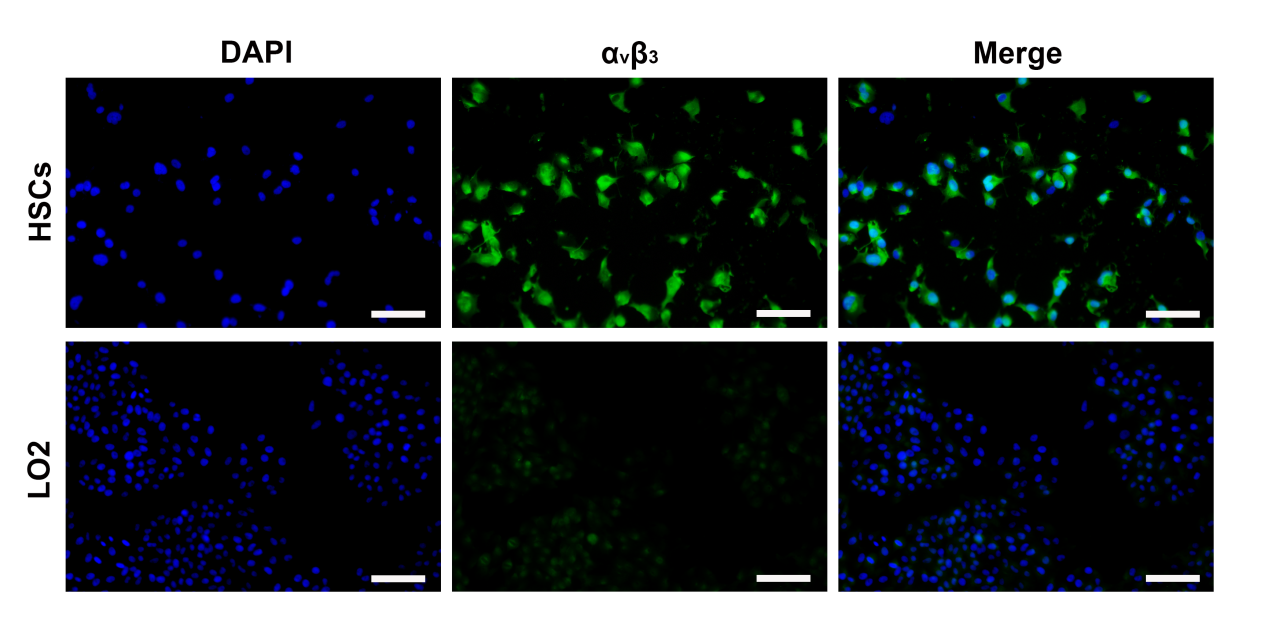


**Figure S4.** Overexpression of α_v_β_3_ against the activated HSCs compared with LO2.


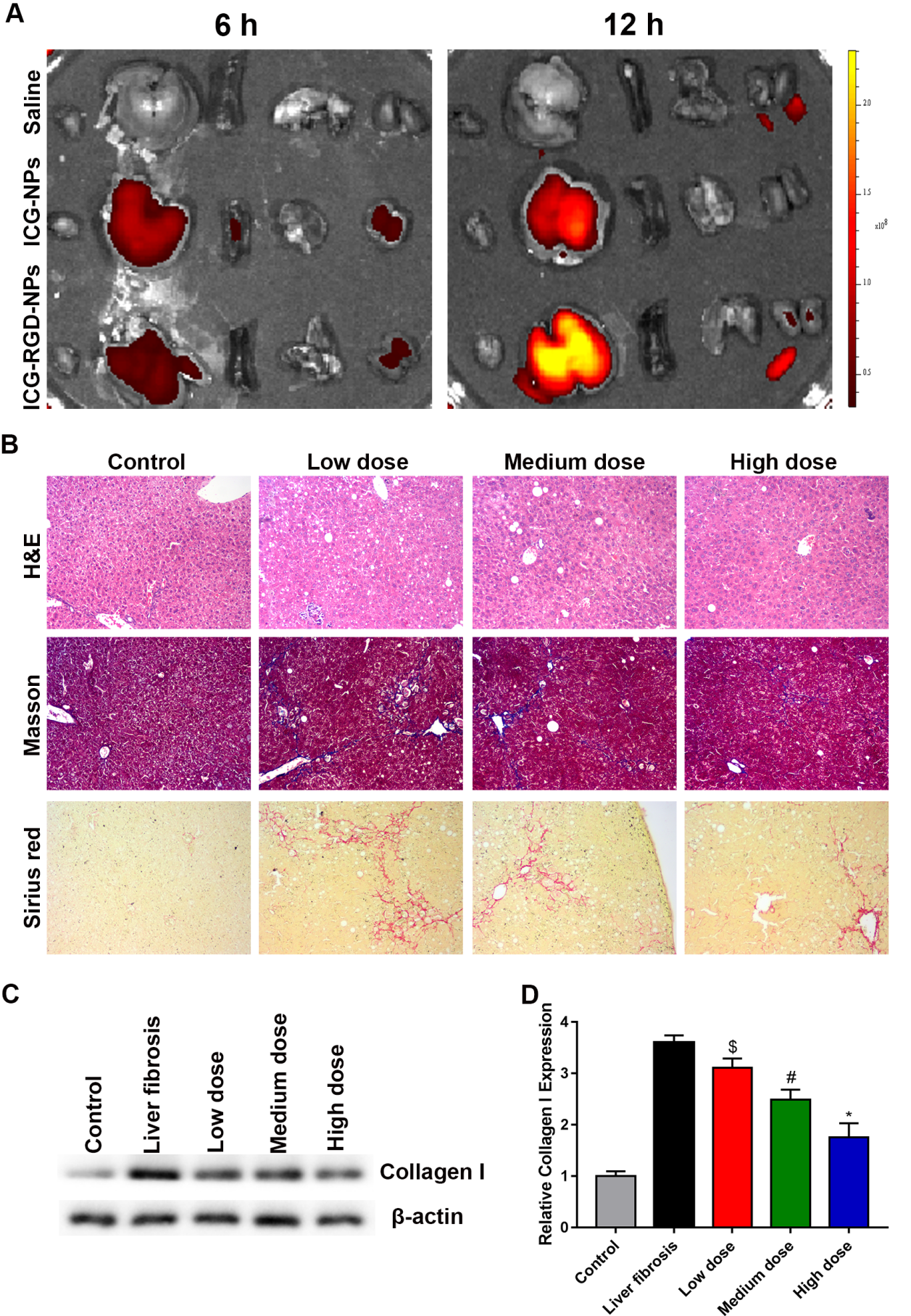


**Figure S5.** Biodistribution of ICG-NPs and ICG-RGD-NPs in mice of liver fibrosis after 6 h and 12 h (A). H&E, Masson and Sirius Red staining (B), collagen I expression (C) and quantitative analysis of collagen I (D) of livers collected from liver fibrotic mice treated with G/R-RGD-NPs of low, medium and high dose (**p* < 0.05 *vs* Medium dose, #*p* < 0.05 *vs* Low dose, $*p* < 0.05 *vs* Liver fibrosis).
